# Transplant Indications in Polycystic Liver and Kidney Disease

**DOI:** 10.5152/tjg.2026.26217

**Published:** 2026-05-20

**Authors:** Sandra Perez-Prado, Abid Suddle

**Affiliations:** King’s College Hospital Institute of Liver Studies, London, UK

**Keywords:** Combined liver and kidney transplantation, liver transplantation, polycystic liver disease

## Abstract

Polycystic liver and kidney disease encompasses a spectrum of inherited disorders ,primarily autosomal dominant polycystic kidney disease (ADPKD) and autosomal dominant polycystic liver disease (ADPLD). Although many patients remain asymptomatic for decades, a subset develops debilitating complications, including massive hepatomegaly, recurrent cyst infections, malnutrition, and progressive renal dysfunction. Diagnosis relies on imaging criteria and genetic testing. ADPKD is most commonly associated with PKD1 and PKD2 mutations, whereas ADPLD is associated with PRKCSH and SEC63 mutations. Magnetic resonance imaging provides optimal assessment of cyst distribution, liver volume, and complications. Disease severity classifications, such as Schnelldorfer classification, guide therapeutic decision-making. Liver transplantation is the only definitive treatment for severe symptomatic PLD. Primary indications include recurrent cyst infections refractory to medical therapy, massive hepatomegaly with significant impairment of quality of life, malnutrition, and portal hypertension. Patient-reported instruments, including PLD-Q and POLCA, questionnaires, provide objective measures of symptom burden and aid in transplant candidacy assessment. In patients with concurrent renal insufficiency (estimated glomerular filtration rate <30 mL/min/1.73 m^2^), combined liver–kidney transplantation may improve outcomes and confer potential immunoprotective benefits for the renal graft. Despite favorable posttransplant outcomes—including 5-year survival rates exceeding 80%—variation in listing criteria persists, highlighting the need for standardized, evidence-based guidelines. Multidisciplinary evaluation and individualized management remain essential to optimize clinical outcomes in this complex patient population.

## Introduction

Polycystic liver and kidney disease (PLKD) refers to a group of genetic disorders inherited in an autosomal dominant manner. Based on the most prevalent phenotype, these disorders are classified into 2 dominant entities: autosomal dominant polycystic kidney disease (ADPKD) and autosomal dominant polycystic liver disease (ADPLD), with ADPKD being the most common entity.

Although most patients remain asymptomatic for years, a subset develops debilitating symptoms due to massive hepatomegaly, compression of adjacent organs, recurrent cyst infections, severe malnutrition, and progressive decline in renal function. Solid organ transplantation is the only curative option for patients with advanced disease and offers excellent long-term survival rates.

### Diagnosis

ADPKD is a genetic disorder characterized by multiple bilateral renal cysts that lead to progressive decline in renal function and are associated with complications such as hypertension and hepatic cysts. PLD is defined by the presence of more than 10 hepatic cysts not connected to the biliary system. More than 90% of cases by mutations in *PKD1* and *PKD2*. Diagnosis is established by genetic testing in patients with a known familial mutation and by imaging criteria (Table 1) in those without an identified familial mutation or in cases of incidental detection.^1^

In patients with ADPLD, disease is confined to the liver with minimal or no renal involvement, and the most frequently associated genes are *PRKCSH* and *SEC63*.

Once PLD is diagnosed, computed tomography (CT) or magnetic resonance imaging (MRI) is required to evaluate cyst distribution, the volume of unaffected liver, and the presence of complications such as portal hypertension. MRI is superior because of its better detection of small cysts. Several classification systems, including those by Giot, Kim, or Schnelldorfer, stratify disease severity based on cyst number, distribution, and size and help guide therapeutic decision-making. The Schnelldorfer classification, for example, differentiates patients who may benefit from resection vs. liver transplantation (LT), and is summarized in Table 2.^2^

Furthermore, in pharmacological clinical trials and in some surgical studies, height-adjusted liver volume is used as an endpoint, even though it does not reflect symptom burden.^3^^-^^11^ Some studies have shown that liver volume does not always correlate with symptoms,^12^ whereas others have reported a moderate correlation between height-adjusted hepatic and renal volume and symptom development.^13^ Therefore, in addition to liver and kidney volume, other factors appear to significantly influence symptom development, such as a history of urinary tract infections, renal or hepatic cyst infections, and hematuria.

The gold standard for measurement is manual tracing, but automated approaches have been developed that strongly correlate with manually traced volumes.^3^

### Clinical Evaluation

Most patients with PLD remain asymptomatic. However, some may develop rapidly progressive disease, particularly premenopausal women. Approximately 20% of patients with ADPKD-associated PLD develop PLD-related symptoms.^14^ Because liver function is typically preserved, even in severe cases, the development of significant symptoms is the primary indication for treatment.

In advanced cases, symptoms are primarily related to progressive abdominal distension secondary to massive hepatomegaly. Common clinical manifestations include early satiety, abdominal pain, abdominal wall hernias, lower back pain, and supine dyspnea. Cyst-related complications may also occur, including rupture, hemorrhage, or infection. Cholestasis secondary to biliary compression may develop. In severe cases, compression of the hepatic or portal veins can lead to portal hypertension, resulting in ascites or hepatic hydrothorax due to impaired venous outflow from the liver to the inferior vena cava (IVC).

### Transplant Criteria

Although LT is the only curative option for patients with PLD, only those with advanced cases are considered candidates. In most countries, LT allocation is based on mortality risk as determined by the model for end-stage liver disease (MELD) score, which has been validated for patients with cirrhosis and end-stage liver disease. As previously mentioned, patients with PLD typically have preserved liver function, even in advanced stages; therefore, the MELD score will not reflect the need for LT.

The European Association for the Study of the Liver clinical practice guidelines recommend evaluation for LT in patients with severe impairment of quality of life, complications treatable only with transplantation, or failure or contraindication of other therapeutic interventions.^14^ Some allocation systems have incorporated exception criteria to improve prioritization. Although this approach facilitates access to LT in patients with PLD, disease-specific criteria vary across European organ allocation systems, and only a limited number have established specific exception policies. This lack of uniform criteria results in practice variability and contributes to differences in LT listing thresholds.

Within Europe, only Spain, the United Kingdom, France, and the countries within the Eurotransplant network have established PLD-specific listing criteria.^15^

In Spain, patients with PLD meeting any of the following criteria are granted MELD exception points (MELD score 19, with an additional 1 point every 3 months):

### Malnutrition

Uncontrolled complications of liver cysts despite medical therapy include recurrent infection despite adequate therapy and Budd–Chiari-like syndrome due to cyst compression.

### Symptomatic Portal Hypertension Due to Polycystic Liver Disease (Ascites or Variceal Bleeding)

In the United Kingdom, the NHS Blood and Transplant also uses PLD-specific selection criteria, including intractable symptoms due to mass of liver or pain unresponsive to cystectomy and severe complications secondary to portal hypertension. Patients are listed on the variant syndrome category due to their preserved liver function.

In France, specifically for PLD, an expert MELD 800-point component can be requested. A panel of medical experts reviews these requests. If approved, the timing for allocation of the maximum points is specified, and patients may receive the maximum score immediately in life-threatening emergencies, or at 3, 6, 9, or 12 months depending on clinical urgency. The 12-month allocation is primarily used for selected pediatric indications.

In the case of countries within Eurotransplant, the listing criteria are as follows:

- Massive PLD (total cysts/parenchyma ratio >1) with complication(s) that can only be treated by LT;
- Clinically significant liver disease due to massive PLD, including weight loss, ascites, and portal hypertension;
- Failure of or contraindications to nontransplant-related interventions; and
- Contraindications to nontransplant-related interventions, fulfilling criteria 1 and 2.

Despite the absence of standardized criteria, European specialists consistently identify several key indications for LT in patients with PLD, as outlined below.

In the United States, the specific LT criteria are as follows:

- PLD with severe symptoms and any 1 or more of the following items:
  Hepatic decompensation or severe portal hypertension complication
  Concurrent hemodialysis
  Glomerular filtration rate <20 mL/min
  Prior kidney transplant
  Moderate-to-severe protein-calorie malnutrition, as documented by a registered dietitian using any of the following:

- (a) Modified Global Leadership Initiative on Malnutrition phenotypic criteria
- (b) American Society for Enteral and Parenteral Nutrition criteria
- (c) Nutrition-Focused Physical Exam
- >(d) Subjective Global Assessment score

- Severe sarcopenia as documented by skeletal muscle index (<39 cm^2^/m^2^ in women and < 50 cm^2^/m^2^ in men) or an equivalent measure.

Candidates meeting the criteria described above should be considered for an MELD exception score equal to the median MELD at transplant.

Transplant outcomes in PLD are generally very good, with comparable 30-day and 1-year patient survival rates and 5-year higher survival rates compared with non-PLD indications (81.3% vs. 76.5%).This occurs despite longer waiting times. However, increased perioperative mortality has been reported, likely due to greater operative complexity in PLD, which may be exacerbated by delayed transplantation and continued cyst and liver growth. A retrospective study from the United Kingdom reported intraoperative mortality of 1% in PLD recipients compared with 0.4% in non-PLD recipients over a 7-year period.^16^

#### Quality of Life:

The main indication for LT is massive hepatomegaly associated with significant impairment of quality of life, which develops in approximately 2%-5% of patients with PLD.^14^ Symptoms include abdominal pain and restriction of mobility and food intake. Disease-specific quality-of-life questionnaires have been developed to better assess symptom burden in this population because generic quality-of-life instruments lack sufficient sensitivity to capture PLD-related symptoms. Moreover, data regarding the correlation between liver volume and symptom severity remain controversial.

The first of these instruments is the PLD-Q, a patient-reported outcome measure consisting of 16 items, with scores ranging from 0 to 100. It was specifically designed to identify PLD-related symptoms and their impact on quality of life.^18^ The PLD-Q has been validated in both European and North American cohorts and is currently used in clinical practice, clinical trials, and observational studies to assess treatment response in patients with PLD.^5^^,19^ Following its development, a treatment threshold was established: patients with a score of 32 or greater are considered candidates for therapeutic intervention. However, no specific cutoffs have been defined to guide the choice of treatment modality.^20^

The second instrument is the POLCA questionnaire, a self-report questionnaire designed and validated to assess the presence and severity of hepatomegaly-related complaints in patients with symptomatic PLD.^21^ It consists of 16 items, each scored from 0 to 5, across 4 subscales: severity of perceived illness (SPI) (score range, 0-35; 7 items); gastroesophageal reflux disease–related complaints (score range, 0-20; 4 items); impact on food intake (score range, 0-10; 2 items); and perception of enlarged liver volume (score range, 0-15; 3 items).

A POLCA SPI score of 14 or greater predicts the need to initiate treatment, whereas patients with a score lower than 7 are unlikely to require therapy.^22^ Furthermore, a POLCA SPI score of 16.5 or greater predicts the need for LT, with a sensitivity of 81.3% and a specificity of 88.9%.^23^

#### Malnutrition:

Malnutrition is one of the most serious complications of PLD and constitutes an indication for referral for LT. Although the specific impact of malnutrition and sarcopenia on LT outcomes has not been extensively studied in patients with PLD, their role as predictors of posttransplant complications and postoperative recovery is well established in the broader transplant literature.^15^

Malnutrition is typically observed in advanced stages of PLD, particularly in patients receiving dialysis. It is mainly related to early satiety, nausea, and vomiting caused by gastric compression secondary to massive hepatomegaly. To ensure timely interventions and improve clinical outcomes in patients with PLD, early assessment of nutritional status is essential, even in the initial stages of the disease.^24^

It is important to note that weight loss is often underestimated in these patients because of the additional weight attributable to hepatomegaly. Therefore, alternative methods should be used to assess sarcopenia, such as measurement of midarm circumference in the nondominant arm (<23.8 cm in men and <23.1 cm in women). However, this method has not been validated specifically in PLD and is subject to significant interobserver variability.^14^^,^^25^

A more objective assessment of sarcopenia can be performed using CT or MRI, which allows the calculation of height-adjusted skeletal muscle mass (cm^2^) and subcutaneous adipose tissue (cm^2^) at the level of the third lumbar vertebra (L3), with lower interobserver variability. Sarcopenia is defined as a reduction in muscle mass of at least 2 standard deviations below the mean of a reference population. The cutoffs commonly used to define severe sarcopenia are derived from a prior oncology cohort and are defined by a skeletal muscle index at L3 <38.5 cm^2^/m^2^ in females and <52.4 cm^2^/m^2^ in males.^26^ Although these thresholds have not been specifically validated in PLD populations, they are widely used in clinical practice and may serve as predictors of patient and graft survival after LT, as sarcopenia has been associated with higher mortality and prolonged intensive care unit stay in transplant recipients.^27^^,^^28^

Finally, the frailty index—an assessment tool incorporating muscle wasting, malnutrition, and functional decline based on grip strength, chair stands, and balance testing—is a well-validated prognostic measure in patients with end-stage liver disease. Its use may also be considered in patients with PLD.^29^

#### Portal Hypertension:

**Figure 1.**
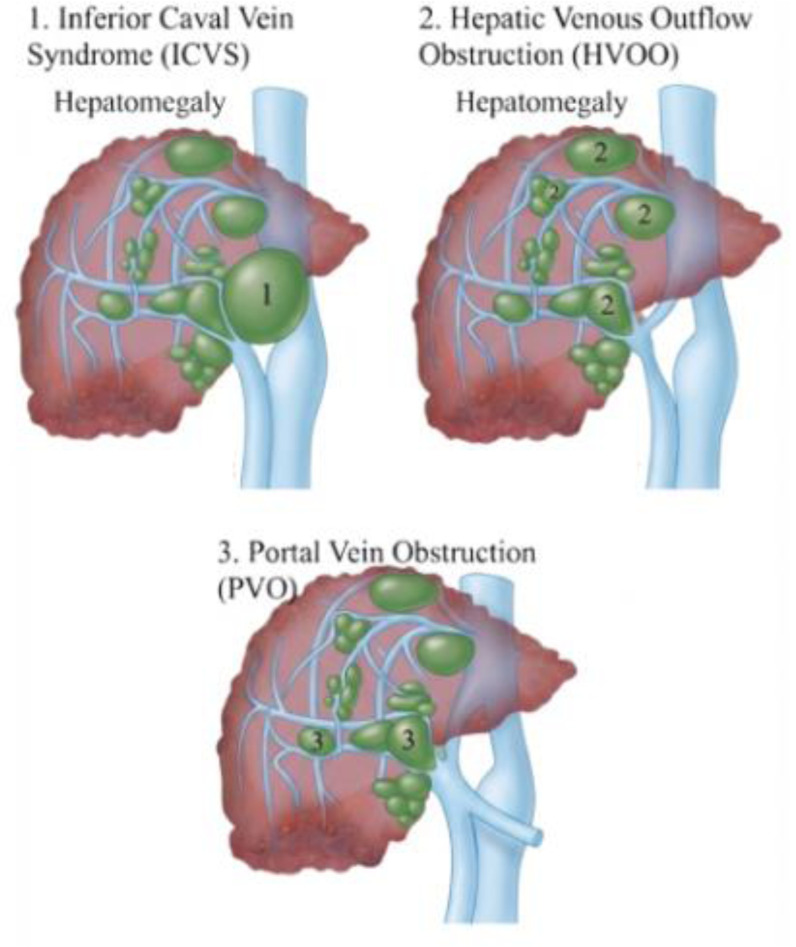
Mechanisms of noncirrhotic portal hypertension.

Clinically significant portal hypertension is a syndrome characterized by splenomegaly, ascites, varices, and hepatic encephalopathy and is defined by an increased hepatic venous pressure gradient. In advanced cases of PLD, clinically significant portal hypertension may also occur. It is primarily caused by 3 forms of vascular obstruction resulting from the mass effect of hepatic cysts: inferior caval vein syndrome, hepatic venous outflow obstruction (HVOO), and portal vein obstruction, as shown in Figure 1. These mechanisms lead to the development of noncirrhotic portal hypertension, with no significant differences according to the underlying etiology of PLD.^30^^,^^31^ Among these, HVOO represents the most common cause of portal hypertension in this population.

Data regarding the incidence of portal hypertension in patients with PLD are limited. A single-center study from the United Kingdom including 47 patients with PLD listed for LT reported that 42.7% had evidence of portal hypertension.^32^

Among the clinical manifestations of portal hypertension, varices are relatively uncommon, with a reported prevalence of approximately 2% among patients listed for LT.^32^ Ascites is the most frequent manifestation, and its development correlates with liver volume^33^ as well as other risk factors, such as prior abdominal surgery.^34^ Postoperative ascites is often transient and typically responds to diuretic therapy; however, persistent ascites may occur in some cases.^35^

LT remains the only curative therapy in these patients and should be considered in those presenting with variceal bleeding or refractory ascites unresponsive to conventional management, including diuretics, liver volume–reducing therapies, and vascular stenting or shunting.

#### Cyst-Related Complications:

Recurrent cyst infections represent a significant indication for LT. Cyst infection is estimated to occur in approximately 5% of patients with PLD, with the risk increasing to 37% in patients with ADPKD and hepatic cysts following kidney transplantation. Once this complication develops, it often requires prolonged hospital admissions and is associated with substantial morbidity and mortality.

LT remains the only definitive treatment for a selected group of patients with chronic cyst infection or infection refractory to medical therapy. In 2024, an expert consensus established that recurrent cyst infection—defined as more than 2 episodes within a 6-month period—should be considered an indication for LT, particularly in patients listed for kidney transplantation.^17^

### Special Considerations

#### Indications for Combined Liver–Kidney Transplantation:

In general, patients with ADPKD who meet the previously described criteria for LT and who also have an estimated glomerular filtration rate <30 mL/min/1.73 m^2^ should be considered for combined liver–kidney transplantation (CLKT).^1^^,^^14^ This threshold does not typically qualify patients for isolated kidney transplantation but serves as a pragmatic cutoff to preempt the need for future dialysis.

Some studies suggest improved survival in patients undergoing CLKT compared with those receiving LT alone, with reported 3- and 5-year survival rates exceeding 90%. The higher perioperative mortality in patients listed for isolated LT may reflect greater surgical complexity of the procedure, as these patients may have been more severe pretransplant complications, such as recurrent cholangitis, esophageal variceal bleeding, and malnutrition.^36^

Regarding the performance of a simultaneous or sequential liver–kidney transplantation, robust data are lacking to determine which strategy is superior. A recent survey among European transplantation center specialists showed that the preferred option was simultaneous transplant (40.4%), followed by liver-first (30.8%), no preference (26.9%), and kidney-first (1.9%).^15^

One argument supporting simultaneous transplantation is the potential immunoprotective effect of the liver allograft on the renal graft when both organs are transplanted together, an effect not observed in sequential kidney transplantation. This phenomenon may reduce the incidence of renal allograft rejection^37^^,^^38^ and could potentially allow for lower levels of immunosuppression compared with isolated kidney transplantation.^39^ Moreover, worsening renal function has been reported following isolated LT;^40^^,^^41^ therefore, simultaneous transplantation may offer the advantage of avoiding renal failure in patients with preexisting cystic kidney disease and may prevent the need for bridging dialysis after LT.

On the other hand, in favor of a sequential transplant strategy—performing LT first—the main argument is that perioperative hemodynamic management differs between patients undergoing LT and those receiving kidney transplantation; therefore, the intraoperative hemodynamic conditions during LT may have a potentially deleterious effect on the kidney graft.^42^^,^^43^ A liver-first approach allows hemodynamic stabilization following LT, thereby minimizing stress on a future kidney graft. Furthermore, the previously described immunological benefit of simultaneous liver–kidney transplantation may be achieved using machine perfusion and delayed implantation of a kidney from the same donor, which offers several advantages, including stabilization of the patient’s hemodynamic condition.^44^

#### Surgical Considerations:

Because of the massive organ enlargement, patients undergoing LT or CLKT for PLD may experience a complex postoperative course. Marked distortion of vascular anatomy is common, making access to the suprahepatic IVC and hepatic veins technically challenging and increasing the risk of intraoperative hemorrhage.

In general, early control of the infrahepatic IVC during the initial dissection phase is discouraged. Instead, dissection of the hepatic hilum is performed first to achieve inflow control. After division of the hepatic artery, additional strategies may include portal venous flow diversion through the creation of a portocaval shunt or the use of full veno-venous bypass.^14^

Surgeons should avoid excessive downward and leftward traction of the liver to prevent tearing of fragile hepatic veins. The liver is progressively mobilized from inferior to superior or from left to right, avoiding leftward rotation, until safe control of the suprahepatic IVC is achieved.

A case series of 32 patients in whom the conventional technique was modified to include routine IVC veno-venous bypass demonstrated high survival rates, consistent with previous reports. Postoperative complications were comparable to those observed in patients undergoing transplantation for other indications, with 5-year survival rates exceeding 80% and significant improvement in quality of life following transplantation.^16^^,^ ^45^^-^^48^

An additional intraoperative consideration is whether to perform native nephrectomy during combined liver–kidney transplantation. Native nephrectomy should be reserved for selected cases in which the potential benefits outweigh the risks. Indications include severe symptoms due to massively enlarged kidneys, recurrent or severe renal infection or bleeding, complicated nephrolithiasis, intractable pain, suspicion of renal cell carcinoma, insufficient space for the renal graft, and severe ventral hernia.^1^

#### Screening for Intracranial Aneurysms:

Patients with ADPKD have an increased risk of intracranial aneurysms and, consequently, subarachnoid hemorrhage compared with the general population, with a reported prevalence of up to 12%.^49^

Female sex, hypertension, a family history of intracranial hemorrhage, and advanced chronic kidney disease significantly increase the likelihood of aneurysm detection. In this population, aneurysms tend to present at a younger age and carry a higher risk of rupture.

Therefore, in high-risk settings such as pretransplant evaluation, screening should be considered. The preferred imaging modality is magnetic resonance angiography without gadolinium enhancement, whereas high-resolution CT angiography may be used as an alternative when appropriate.^1^

## Conclusion

PLKD represents a complex therapeutic challenge that requires a multidisciplinary and individualized approach. LT is the only curative option for severe symptomatic PLD, with indications including recurrent cyst infections, significant deterioration in quality of life, and severe malnutrition. Outcomes of LT in this population are excellent, with 5-year survival rates exceeding 80%.

Despite these excellent clinical outcomes, significant variability persists in selection criteria and access to transplantation among different centers and countries, highlighting the need to establish unified, evidence-based criteria to ensure equitable access to these potentially curative therapies.

## Figures and Tables

**Table 1.** Kidney Ultrasound Criteria for ADPKD Diagnosis

Diagnosis confirmed	Diagnosis ruled out
≥3 total cysts present at ages 15-39	≤1 cyst present at ages 15-39
≥2 cysts present in each kidney at ages 40-59	≤2 cysts total at ages 40-59
≥4 cysts present in each kidney over 60 years	

**Table 2.** Summary of Schnelldorfer’s Classification That Aims at Differentiating Patients Who Could Benefit from Resection or Transplantation

	Type A	Type B	Type C	Type D
Symptoms	Absent or mild	Moderate to severe	Severe (or moderate)	Severe (or moderate)
Cyst characteristics	Any	Limited number of large cysts	Any	Any
Areas of relative normal liver parenchyma	Any	2 or more sectors	1 or more sectors	Less than 1 sector
Presence of portal vein or hepatic vein occlusion in the preserved hepatic sectors	Any	Absent	Absent	Present
Recommend therapy	Observation or medical therapy	Cyst fenestration	Partial hepatectomy with possible fenestration of remnant cysts	Liver transplantation

## Data Availability

The data that support the findings of this study are available on request from the corresponding author.

## References

[1] Torres VE Ahn C Barten TRM KDIGO 2025 clinical practice guideline for the evaluation, management, and treatment of autosomal dominant polycystic kidney disease (ADPKD): executive summary. Kidney Int. 2025;107(2):234 254. (doi: 10.1016/j.kint.2024.07.010) 39848746

[2] Abu-Wasel B Walsh C Keough V Molinari M. Pathophysiology, epidemiology, classification and treatment options for polycystic liver diseases. World J Gastroenterol. 2013;19(35):5775 5786. (doi: 10.3748/wjg.v19.i35.5775) 24124322 PMC3793132

[3] Van Gastel MDA Edwards ME Torres VE Erickson BJ Gansevoort RT Kline TL. Automatic measurement of kidney and liver volumes from MR images of patients affected by autosomal dominant polycystic kidney disease. J Am Soc Nephrol. 2019;30(8):1514 1522. (doi: 10.1681/ASN.2018090902) 31270136 PMC6683702

[4] van Keimpema L Nevens F Vanslembrouck R Lanreotide reduces the volume of polycystic liver: a randomized, double-blind, placebo-controlled trial. Gastroenterology. 2009;137(5):e1661-1662. (doi: 10.1053/j.gastro.2009.07.052) 19646443

[5] Bernts LHP Neijenhuis MK Edwards ME Symptom relief and quality of life after combined partial hepatectomy and cyst fenestration in highly symptomatic polycystic liver disease. Surgery. 2020;168(1):25 32. (doi: 10.1016/j.surg.2020.02.014) 32402542 PMC7347464

[6] van Keimpema L Ruurda JP Ernst MF van Geffen HJAA Drenth JPH. Laparoscopic fenestration of liver cysts in polycystic liver disease results in a median volume reduction of 12.5%. J Gastrointest Surg. 2008;12(3):477 482. (doi: 10.1007/s11605-007-0376-8) 17957434

[7] Hogan MC Masyuk TV Page LJ Randomized clinical trial of long-acting somatostatin for autosomal dominant polycystic kidney and liver disease. J Am Soc Nephrol. 2010;21(6):1052 1061. (doi: 10.1681/ASN.2009121291) 20431041 PMC2900957

[8] Caroli A Antiga L Cafaro M Reducing polycystic liver volume in ADPKD: effects of somatostatin analogue octreotide. Clin J Am Soc Nephrol. 2010;5(5):783 789. (doi: 10.2215/CJN.05380709) 20185596 PMC2863977

[9] Pisani A Sabbatini M Imbriaco M Long-term effects of octreotide on liver volume in patients with polycystic kidney and liver disease. Clin Gastroenterol Hepatol. 2016;14(7):1022 1030.e4. (doi: 10.1016/j.cgh.2015.12.049) 26844873

[10] van Aerts RMM Kievit W D’Agnolo HMA Lanreotide reduces liver growth in patients with autosomal dominant polycystic liver and kidney disease. Gastroenterology. 2019;157(2):481 491.e7. (doi: 10.1053/j.gastro.2019.04.018) 31022403

[11] Hogan MC Chamberlin JA Vaughan LE Pansomatostatin agonist pasireotide long-acting release for patients with autosomal dominant polycystic kidney or liver disease with severe liver involvement: a randomized clinical trial. Clin J Am Soc Nephrol. 2020;15(9):1267 1278. (doi: 10.2215/CJN.13661119) 32843370 PMC7480539

[12] Wijnands TFM Neijenhuis MK Kievit W Evaluating health-related quality of life in patients with polycystic liver disease and determining the impact of symptoms and liver volume. Liver Int. 2014;34(10):1578 1583. (doi: 10.1111/liv.12430) 24313956

[13] D’Agnolo HMA Casteleijn NF Gevers TJG The association of combined total kidney and liver volume with pain and gastrointestinal symptoms in patients with later stage autosomal dominant polycystic kidney disease. Am J Nephrol. 2017;46(3):239 248. (doi: 10.1159/000479436) 28881341

[14] Drenth J Barten T Hartog H EASL clinical practice guidelines on the management of cystic liver diseases. J Hepatol. 2022;77(4):1083 1108. (doi: 10.1016/j.jhep.2022.06.002) 35728731

[15] Duijzer R Alwayn IPJ Ferri F Liver transplantation indications and strategies in polycystic liver disease: a European survey. Liver Transpl. 2025. (doi: 10.1097/LVT.0000000000000700) PMC12991327 40793999

[16] Gittus M Moore J Ong ACM. Liver transplant recipients with polycystic liver disease have longer waiting times but better long-term clinical outcomes than those with liver disease due to other causes: a retrospective cross-sectional study. PLOS One. 2024;19(1):e0294717. (doi: 10.1371/journal.pone.0294717) PMC10760649 38165905

[17] Duijzer R Bernts LHP Geerts A Clinical management of liver cyst infections: an international, modified Delphi-based clinical decision framework. Lancet Gastroenterol Hepatol. 2024;9(9):884 894. (doi: 10.1016/S2468-1253(24)00094-3) 38878785

[18] Neijenhuis MK Gevers TJG Hogan MC Development and validation of a disease-specific questionnaire to assess patient-reported symptoms in polycystic liver disease. Hepatology. 2016;64(1):151 160. (doi: 10.1002/hep.28545) 26970415 PMC4917464

[19] Neijenhuis MK Kievit W Verheesen SM D’Agnolo HM Gevers TJ Drenth JP. Impact of liver volume on polycystic liver disease–related symptoms and quality of life. U Eur Gastroenterol J. 2018;6(1):81 88. (doi: 10.1177/2050640617705577) PMC5802666 29435317

[20] Barten TRM Staring CB Hogan MC Gevers TJG Drenth JPH. Expanding the clinical application of the polycystic liver disease questionnaire: determination of a clinical threshold to select patients for therapy. HPB. 2023;25(8):890 897. (doi: 10.1016/j.hpb.2023.04.004) 37095030

[21] Temmerman F Dobbels F Ho TA Development and validation of a polycystic liver disease complaint–specific assessment (POLCA). J Hepatol. 2014;61(5):1143 1150. (doi: 10.1016/j.jhep.2014.06.024) 24996047

[22] Billiet A Temmerman F Coudyzer W Questionnaire PLD-complaint-specific assessment identifies need for therapy in polycystic liver disease: a multi-centric prospective study. U Eur Gastroenterol J. 2023;11(7):633 641. (doi: 10.1002/ueg2.12387) PMC10493353 37278135

[23] Billiet A Moubax K Libbrecht L Van Moerkercke W A yellow sign indicating danger ahead . Acta Gastroenterol Belg . 2022 ; 85 ( 4 ): 654 - 655 . ( https://doi:10.51821/85.3.10922 ) 36566379 10.51821/85.3.10922

[24] Ryu H Kim H Park HC Total kidney and liver volume is a major risk factor for malnutrition in ambulatory patients with autosomal dominant polycystic kidney disease. BMC Nephrol. 2017;18(1):22. (doi: 10.1186/s12882-016-0434-0) PMC5237538 28088190

[25] Arrazola L Moonka D Gish RG Everson GT. Model for end-stage liver disease (MELD) exception for polycystic liver disease. Liver Transpl. 2006;12(suppl 3):S110 S111. (doi: 10.1002/lt.20974) 17123287

[26] Tandon P Ney M Irwin I Severe muscle depletion in patients on the liver transplant wait list: its prevalence and independent prognostic value. Liver Transpl. 2012;18(10):1209 1216. (doi: 10.1002/lt.23495) 22740290

[27] Lim M Kim JM Yang J Upper thigh skeletal muscle index predicts outcomes in liver transplant recipients. Ann Surg Treat Res. 2023;105(4):219 227. (doi: 10.4174/astr.2023.105.4.219) . 37908380 PMC10613820

[28] Bot D Klerks S Leistra E Tushuizen ME van Hoek B. Association between skeletal muscle index prior to liver transplantation and 1-year mortality posttransplant. J Parenter Enteral Nutr. 2023;47(7):867 877. (doi: 10.1002/jpen.2508) 37070816

[29] Lai JC Covinsky KE Dodge JL Development of a novel frailty index to predict mortality in patients with end-stage liver disease. Hepatology. 2017;66(2):564 574. (doi: 10.1002/hep.29219) 28422306 PMC5519430

[30] Bernts LHP Drenth JPH Tjwa ETTL. Management of portal hypertension and ascites in polycystic liver disease. Liver Int. 2019;39(11):2024 2033. (doi: 10.1111/liv.14245) 31505092 PMC6899472

[31] de Franchis R Baveno VF. Revising consensus in portal hypertension: report of the Baveno V consensus workshop on methodology of diagnosis and therapy in portal hypertension. J Hepatol. 2010;53(4):762 768. (doi: 10.1016/j.jhep.2010.06.004) 20638742

[32] Rajoriya N Tripathi D Leithead JA Portal hypertension in polycystic liver disease patients does not affect wait-list or immediate post-liver transplantation outcomes. World J Gastroenterol. 2016;22(45):9966 9973. (doi: 10.3748/wjg.v22.i45.9966) 28018103 PMC5143763

[33] Kim H Park HC Ryu H Clinical correlates of mass effect in autosomal dominant polycystic kidney disease. PLOS One. 2015;10(12):e0144526. (doi: 10.1371/journal.pone.0144526) PMC4671651 26641645

[34] Bernts LHP Echternach SG Kievit W Rosman C Drenth JPH. Clinical response after laparoscopic fenestration of symptomatic hepatic cysts: a systematic review and meta-analysis. Surg Endosc. 2019;33(3):691 704. (doi: 10.1007/s00464-018-6490-8) 30334152 PMC6394680

[35] Aussilhou B Douflé G Hubert C Extended liver resection for polycystic liver disease can challenge liver transplantation. Ann Surg. 2010;252(5):735 743. (doi: 10.1097/SLA.0b013e3181fb8dc4) 21037428

[36] Coquillard C Berger J Daily M Combined liver-kidney transplantation for polycystic liver and kidney disease: analysis from the United Network for Organ Sharing dataset. Liver Int. 2016;36(7):1018 1025. (doi: 10.1111/liv.13041) 26663575

[37] Simpson N Cho YW Cicciarelli JC Selby RR Fong TL. Comparison of renal allograft outcomes in combined liver-kidney transplantation versus subsequent kidney transplantation in liver transplant recipients: analysis of UNOS Database. Transplantation. 2006;82(10):1298 1303. (doi: 10.1097/01.tp.0000241104.58576.e6) 17130778

[38] Martin EF Huang J Xiang Q Klein JP Bajaj J Saeian K. Recipient survival and graft survival are not diminished by simultaneous liver-kidney transplantation: an analysis of the United Network for Organ Sharing database. Liver Transpl. 2012;18(8):914 929. (doi: 10.1002/lt.23440) 22467623 PMC3405201

[39] Chen X Lu Y Wei L Outcomes of combined liver-kidney transplantation in polycystic liver and kidney disease. Ann Transplant. 2025;30:e947639. (doi: 10.12659/AOT.947639) PMC12049081 40296340

[40] Jeyarajah DR Gonwa TA Testa G Liver and kidney transplantation for polycystic disease. Transplantation. 1998;66(4):529 532. (doi: 10.1097/00007890-199808270-00019) 9734499

[41] Nayagam JS Nawaz A Ramos K Renal dysfunction after liver transplantation for polycystic liver disease. Liver Transpl. 2022;28(10):1674 1677. (doi: 10.1002/lt.26516) 35633077

[42] Wagener G Bezinover D Wang C Fluid management during kidney transplantation: a consensus statement of the Committee on Transplant Anesthesia of the American Society of Anesthesiologists. Transplantation. 2021;105(8):1677 1684. (doi: 10.1097/TP.0000000000003581) 33323765

[43] Morkane CM Sapisochin G Mukhtar AM Perioperative fluid management and outcomes in adult deceased donor liver transplantation—a systematic review of the literature and expert panel recommendations. Clin Transplant. 2022;36(10):e14651. (doi: 10.1111/ctr.14651) 35304919

[44] Kim JY Kim HB Kim JM Immunoprotective effect of liver allograft on patients with combined liver and kidney transplantation. Ann Transplant. 2024;29:e942763. (doi: 10.12659/AOT.942763) PMC10858615 38319291

[45] Rodríguez-Aguilar EF Sastre L Colmenero J Liver and kidney transplantation in polycystic liver and kidney disease. Gastroenterol Hepatol (NY). 2021;44(8):552 558. (doi: 10.1016/j.gastrohep.2020.12.004) 33548353

[46] Burra P Burroughs A Graziadei I EASL clinical practice guidelines: liver transplantation. J Hepatol. 2016;64(2):433 485. (doi: 10.1016/j.jhep.2015.10.006) 26597456

[47] van Keimpema L Nevens F Adam R Excellent survival after liver transplantation for isolated polycystic liver disease: an European Liver Transplant Registry study. Transpl Int. 2011;24(12):1239 1245. (doi: 10.1111/j.1432-2277.2011.01360.x) 21955068

[48] Gedaly R Guidry P Davenport D Peri-operative challenges and long-term outcomes in liver transplantation for polycystic liver disease. HPB. 2013;15(4):302 306. (doi: 10.1111/j.1477-2574.2012.00579.x) 23458516 PMC3608985

[49] Nguyen BA Halpin B Olson V Risk factors for unruptured intracranial aneurysms in asymptomatic patients with autosomal dominant polycystic kidney disease: who needs screening? A systematic review and meta-analysis. J Neurol Surg. 2025;143(1):220 231. (doi: 10.3171/2024.9.JNS241175) 39951701

